# A Novel Chip for Cyclic Stretch and Intermittent Hypoxia Cell Exposures Mimicking Obstructive Sleep Apnea

**DOI:** 10.3389/fphys.2016.00319

**Published:** 2016-07-29

**Authors:** Noelia Campillo, Ignasi Jorba, Laura Schaedel, Blai Casals, David Gozal, Ramon Farré, Isaac Almendros, Daniel Navajas

**Affiliations:** ^1^Unitat de Biofísica i Bioenginyeria, Facultat de Medicina, Universitat de BarcelonaBarcelona, Spain; ^2^Cellular and Respiratory Biomechanics, Institute for Bioengineering of CataloniaBarcelona, Spain; ^3^Centro de Investigación Biomédica en Red de Enfermedades RespiratoriasMadrid, Spain; ^4^Biological Sciences Division, Department of Pediatrics, Pritzker School of Medicine, The University of ChicagoChicago, IL, USA; ^5^Institut d'Investigacions Biomèdiques August Pi i SunyerBarcelona, Spain

**Keywords:** obstructive sleep apnea, intermittent hypoxia, cell stretch, hypoxia-inducible factor, lab-on-a-chip

## Abstract

Intermittent hypoxia (IH), a hallmark of obstructive sleep apnea (OSA), plays a critical role in the pathogenesis of OSA-associated morbidities, especially in the cardiovascular and respiratory systems. Oxidative stress and inflammation induced by IH are suggested as main contributors of end-organ dysfunction in OSA patients and animal models. Since the molecular mechanisms underlying these *in vivo* pathological responses remain poorly understood, implementation of experimental *in vitro* cell-based systems capable of inducing high-frequency IH would be highly desirable. Here, we describe the design, fabrication, and validation of a versatile chip for subjecting cultured cells to fast changes in gas partial pressure and to cyclic stretch. The chip is fabricated with polydimethylsiloxane (PDMS) and consists of a cylindrical well-covered by a thin membrane. Cells cultured on top of the membrane can be subjected to fast changes in oxygen concentration (equilibrium time ~6 s). Moreover, cells can be subjected to cyclic stretch at cardiac or respiratory frequencies independently or simultaneously. Rat bone marrow-derived mesenchymal stem cells (MSCs) exposed to IH mimicking OSA and cyclic stretch at cardiac frequencies revealed that hypoxia-inducible factor 1α (HIF-1α) expression was increased in response to both stimuli. Thus, the chip provides a versatile tool for the study of cellular responses to cyclical hypoxia and stretch.

## Introduction

Obstructive sleep apnea (OSA) is a highly prevalent disorder affecting 3–17% of the adult population (Peppard et al., 2013). This disease is characterized by recurrent total or partial collapse of the upper airway, resulting in periodic blood oxygen desaturations and intermittent hypoxia (IH) at the tissue level. During the last decade, adverse outcomes in cardiovascular, cognitive and metabolic diseases, and cancer have emerged in response to IH from studies carried out in OSA patients and animal models (Vijayan, 2012; Almendros et al., 2013). However, the presence of multiple co-existing confounding factors such as obesity, diabetes, age, sex, or genotypic variance in OSA patients has masked the potential validity of the findings, and further highlighted the importance of developing *in vitro* models to investigate potential mechanisms underlying the inherent consequences of IH at the cellular level (Al Lawati et al., 2009).

Most of the deleterious consequences of OSA, particularly those affecting the cardiovascular system, are exacerbated by oxidative stress and inflammatory cascades associated with the activation of the hypoxia-inducible factor 1 (HIF-1) signaling pathway (Peng et al., 2006; Yuan et al., 2008; Belaidi et al., 2009; He et al., 2014). This transcription factor is a heterodimer composed of an oxygen-regulated α subunit (HIF-1α) and a constitutively expressed β subunit (HIF-1β). In normoxia, the α subunit is hydroxylated by prolyl hydroxylases (PHDs) and targeted for degradation via the ubiquitin-proteasome pathway. Under hypoxia, PHDs activity is inhibited, allowing for increased HIF-1α translocation into the nucleus where it dimerizes with the β subunit (Ke and Costa, 2006; Semenza, 2012) and triggers transcription of a large number of relevant genes (Liu et al., 2012). Even though HIF-1α induction was initially described in the context of cellular responses to reduced oxygen tension, its activation is not restricted to hypoxia (Dery et al., 2005; Kuschel et al., 2012). Biomechanical studies revealed that HIF-1α is also upregulated in cells and tissues exposed to stretch (Kim et al., 2002; Milkiewicz et al., 2007; Lim et al., 2011), leading to similar cellular responses to those triggered by hypoxia (Milkiewicz et al., 2007; Leong et al., 2012). This is particularly relevant since, in OSA, respiratory, and cardiovascular cells are subjected to concomitant IH and mechanical stretch. Remarkably, the aorta and large arteries of patients suffering from OSA are subjected to IH and heart-rate cyclic deformations. Moreover, lung parenchymal cells of these patients are exposed to the combined effect of IH and cyclic stretch caused by breathing efforts. Even though the majority of the work involving IH has primarily focused on its deleterious consequences, hypoxia—via activation of the HIF-1 pathway—could also contribute to enhance mechanisms repairing the injured cardiovascular system. Indeed, published data support the notion that the hypoxic stimulus could mobilize mesenchymal stem cells (MSCs) and other progenitor cells from the bone marrow (Carreras et al., 2009; Rey et al., 2009; Gharib et al., 2010; Vertelov et al., 2013; Zan et al., 2015).

A considerable number of *in vitro* studies have reported on the effect of either isolated cyclic stretch or IH (Ryan et al., 2005; Yuan et al., 2008; Leong et al., 2012; Berger et al., 2013; Chen et al., 2014). However, whether simultaneous application of these stimuli may play an additive or synergistic role remains to be elucidated due to the lack of an experimental system that enables for integration of both rapidly cycling IH along with controlled mechanical stretch. Although in moderate to severe OSA patients IH is characterized by short cycling periods of hypoxia and re-oxygenation (Ruehland et al., 2009; Lloberes et al., 2011), the patterns of experimental IH employed thus far have varied greatly across researchers (Almendros et al., 2014). Moreover, most of the *in vitro* cell studies employed cycles of several minutes to hours, owing to limitations of gas diffusion through the culture medium (Gozal et al., 2005; Ryan et al., 2005; Polotsky et al., 2010; Chen et al., 2014). To circumvent this problem, some researchers developed new experimental approaches (Baumgardner and Otto, 2003; Oppegard et al., 2009; Lo et al., 2012; Tsapikouni et al., 2012; Polak et al., 2015), but only a minority have been able to implement oxygen partial pressure (P_O2_) changes at the cell level in a time scale of seconds. Furthermore, these systems are fraught with several drawbacks that limit their widespread implementation in other laboratories (i.e., complex mechanic and electronic design, constant fluid exchange, incompatibility with high-resolution live cell imaging). To the best of our knowledge, no current device enables the combination of IH and cyclic stretch. The design of a new experimental system to overcome these limitations would therefore offer opportunities to improve our knowledge on the mechanisms driving the deleterious and repairing effects of IH on the large number of different cells implicated in the morbidities of OSA.

To investigate the simultaneous effect of IH and stretch on cell behavior, we designed and characterized a versatile polydimethylsiloxane (PDMS) chip that allows for precisely controlled exposures of cultured cells to high-frequency hypoxia/re-oxygenation cycles and to cyclic stretch. We used the new chip to study the effects of both IH and cyclic stretch stimuli on HIF-1α expression in rat bone marrow-derived MSCs.

## Materials and methods

### Chip design

**Figure 1:**
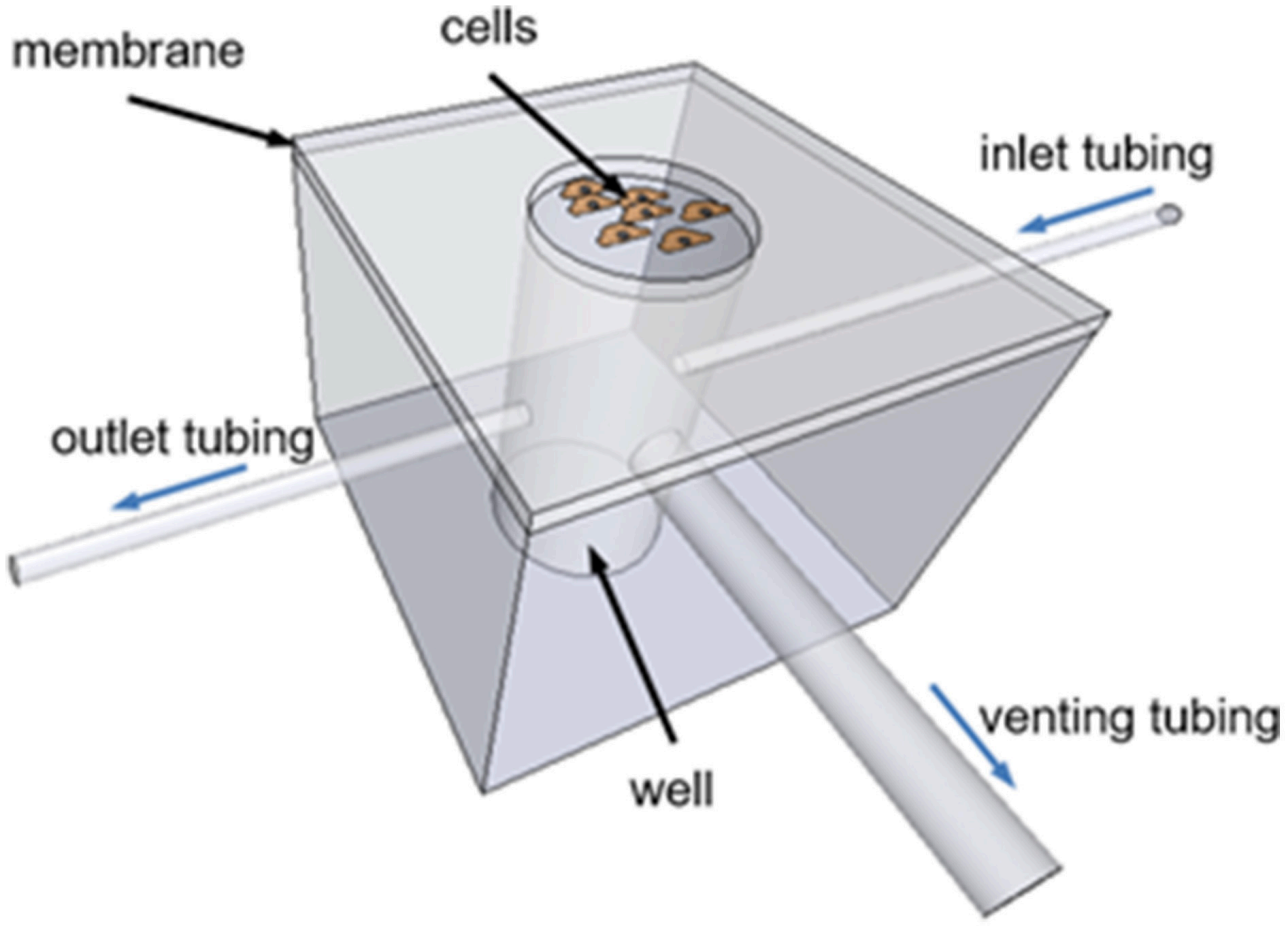
**Schematic drawing of the chip designed to apply intermittent hypoxia and stretch to cultured cells**. The chip consists of a PDMS well-covered with a thin elastic PDMS membrane. Cells are seeded onto the circular clamped membrane. An inlet tubing connects the well to a gas source (not shown) providing a constant mixture of gases or cyclic changes in gas composition. A high resistance outlet tubing increases well-pressure deflecting upwards the membrane. A low resistance venting tubing connects the well to the atmosphere through a cyclically activated two-way solenoid valve (not shown) to produce cyclic membrane stretch.

The chip consists of a cylindrical PDMS well-covered with a thin PDMS membrane (Figure 1). PDMS was chosen for device fabrication due to its biocompatibility, elasticity, high gas permeability, and optical transparency. Cells are cultured on top of the circular clamped membrane coated with extracellular matrix (ECM) proteins. The inlet of the well is connected to a gas source providing a constant mixture of gases or cyclic changes in gas composition. A high resistance outlet tubing open to the atmosphere is connected to the well to inflate the membrane by increasing the pressure inside the well. The well is also open to the atmosphere through a low resistance venting tubing connected to a two-way solenoid valve. Cyclic activation of this valve produces pressure oscillations inside the well-resulting in cyclic membrane stretching. For static experiments, the system can be simplified by replacing the high resistance outlet tubing and the cyclically occluded venting tubing by a single low resistance outlet tubing to maintain well-pressure close to the atmospheric values.

### Chip fabrication

PDMS wells were produced with a 10:1 mixture of pre-polymer and curing agent (Sylgard 184 kit, Dow Corning, MI; see Supplementary Video). The mixture was poured to a thickness of 8 mm onto a culture dish (Techno Plastic Products AG, Trasadingen, Switzerland). The PDMS was degassed for 45 min in a bell jar vacuum desiccator (Kartell Labware, Noviglio, Italy) to avoid bubble formation and baked in an oven (Selecta, Barcelona, Spain) at 65°C for 3 h. After curing, the PDMS was cut into rectangular (~10 × 10 mm) blocks using a scalpel. The PDMS blocks were perforated from top to bottom with a 4 mm diameter punch (Harris Uni-Core, Ted Pella, Redding, CA) to produce a central well. The blocks were also laterally punched to connect inlet, outlet, and venting tubing.

For PDMS membrane fabrication, a 76 × 26 mm glass slide (Deltalab, Chalgrove, UK) was cleaned by gentle agitation in acetone, methanol, and isopropyl alcohol for 30 s in a sequential manner, removing organic contaminants. Then, the slide was N_2_-dried and heated on a hot plate (Selecta) at 95°C for 20 min for complete dehydratation. The glass surface was activated through oxygen plasma treatment using a plasma cleaner (PDC-002, Harrick Scientific Products Inc., Pleasantville, NY) at the highest power (18 W) for 30 s. Finally, the slide was exposed to Repel Silane [(Tridecafluoro-1,1,2,2-tetrahydrooctyl) trichlorosilane] (ABCR GmbH & Co. KG, Karlsruhe, Germany) vapors in the vacuum desiccator for 1 h. A 10:1 PDMS degassed mixture was poured on the slide and spun (Laurell Technologies Corporation, North Wales, PA) at 2000 RPM for 5 s followed by a step of 5000 RPM for 60 s to obtain a thickness of ~10 μm. After spinning, the slide was baked on the hot plate at 95°C for 20 min to cure the PDMS. Finally, membrane thickness was measured using a profilometer (Veeco Instruments, Plainview NY). Alternatively, commercially available PDMS membranes with a nominal thickness of 37.5 μm (Gel-Pak, Hayward, CA) were also used to build the chip. The stiffness of both membranes was measured by Atomic Force Microscopy (AFM). Briefly, the membranes were placed in a custom-built AFM (Alcaraz et al., 2003) and force-indentation curves were obtained with a V-shape Au-coated cantilever with a four-sided pyramidal tip on its apex (MLCT, Bruker, Mannheim, Germany). Membrane Young's modulus (E) was computed by fitting the force-indentation curve with the Hertz contact model (Alcaraz et al., 2003).

**Figure 2:**
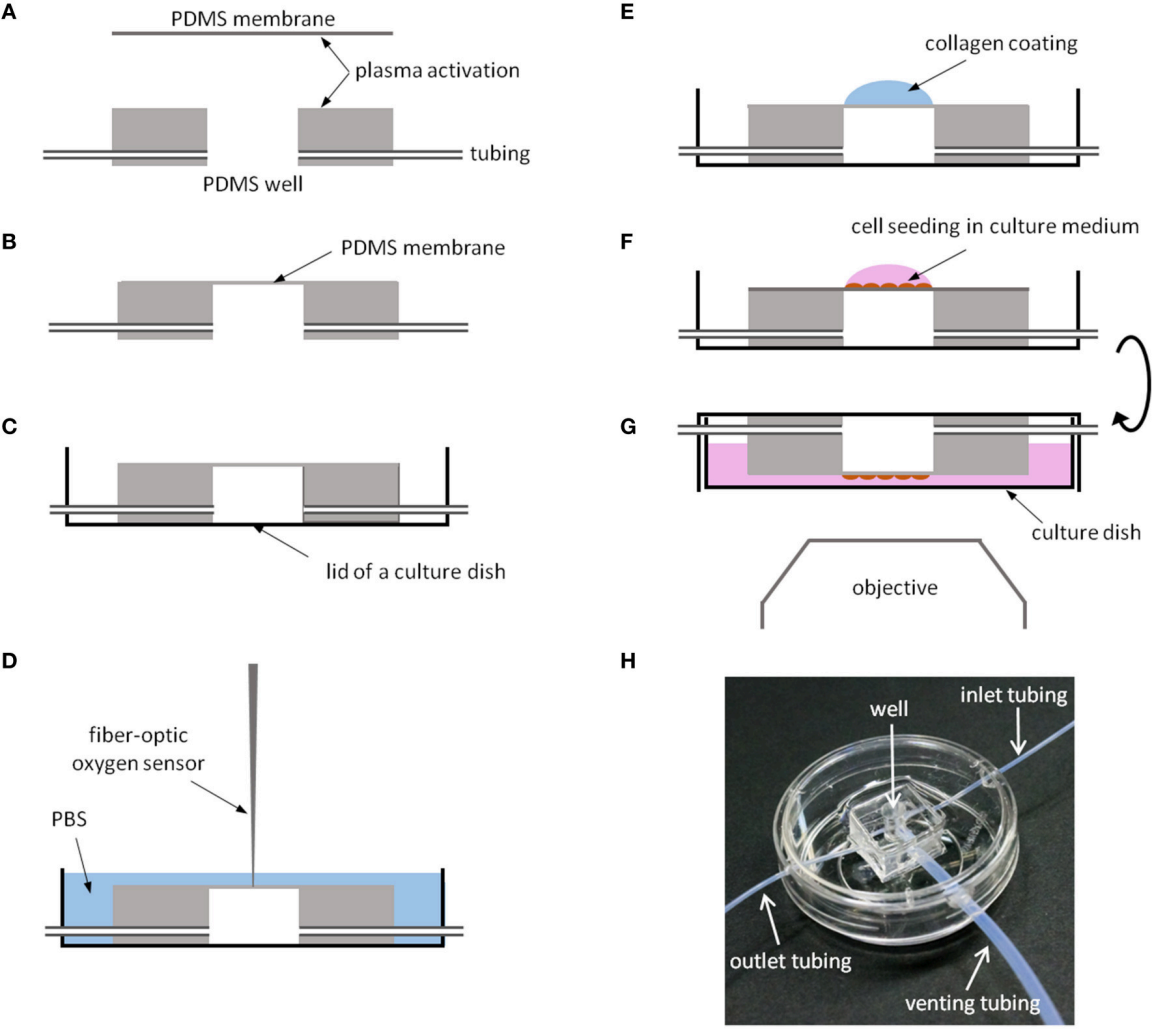
**Chip fabrication. (A)** One surface of a thin PDMS membrane and a PDMS well are activated with plasma to promote PMDS bonding. **(B)** PDMS chip. **(C)** The chip is attached with PDMS to the lid of a culture dish. **(D)** Oxygen measurements at the cell culture level using a fiber-optic O_2_ sensor. **(E)** The region of the membrane covering the well is functionalized with collagen I. **(F)** Cell seeding onto the collagen-coated membrane. **(G)** The culture dish is filled with medium and the chip is inverted to improve cell imaging. **(H)** PDMS chip with a 4 mm ID well 8 mm in depth placed in a culture dish.

To fabricate the chips (Figure 2), one of the surfaces of a PDMS membrane and a well were activated with plasma using a low-cost portable corona treater (Electro Technic Products, Chicago, IL) at close proximity (~5 mm) at the highest voltage: 30 s for the membrane and 1 min for the well (Haubert et al., 2006). The activated side of the well was pressed against the activated side of the membrane. The edges of the membrane were cut using a scalpel and the chip was carefully peeled off from the silanized glass slide. To improve cell culture imaging in an inverted microscope, the base of the chip was adhered to the lid of a 35 mm glass bottom culture dish (MatTek Corporation, Ashland MA) with PDMS mixture placed around its edges and cured in the oven at 65°C for 2 h. Otherwise, image quality would be reduced due to the presence of the PDMS membrane between the cells and the microscope objective. Inlet, outlet, and venting tubes (Cole Parmer, Vernon Hills, IL) were connected to the chip and the wall of the culture dish was drilled at its edge to allow the passage of tubes.

An inlet tubing of 0.3 mm ID (~1 m long) was connected to a servocontrolled gas blender (Gas Blender 100 Series, MCQ Instruments, Rome, Italy) able to provide fast changes in gas composition. Alternatively, gas changes were also generated by switching between two gas sources by means of a cyclically activated three-way solenoid valve (Sirai, Milan, Italy). Cyclic strain of the membrane was produced by increasing the pressure inside the well by a high resistance outlet tubing of 0.3 mm ID and periodic closure of the low resistance venting tubing 1.3 mm ID (~80 cm long) with a two-way solenoid valve (Sirai). The length of the outlet tube was adjusted to provide the desired level of strain. Chips with only the inlet tubing and a low resistance outlet tubing (0.8 mm ID, 4 cm long) without solenoid valve were also fabricated for normoxia, IH, and sustained hypoxia (SH) experiments performed with no cell stretching.

A more detailed protocol can be found in Supplementary Material (Data Sheet 1).

### Assessment of gas equilibrium kinetics through the membrane

Two independent methods were used to measure P_O2_ changes at the cell culture level during application of intermittent hypoxia patterns (0–20% O_2_, 30 s each condition) to chips attached to the bottom of a culture dish covered with 1 × PBS. Rise time of P_O2_ change was computed as the time required for P_O2_ to rise from 10 to 90% of its final change. The first method used a fiber-optic oxygen meter (FireSting O_2_, Pyro Science, Aachen, Germany, nominal response time < 0.3 s) placed above the surface of the membrane at the cell culture level. Prior to experiments, the sensor was calibrated following manufacturer's instructions using two reference points: air saturated with water (21% O_2_) and an anoxic solution of 0.1 M sodium ascorbate and NaOH. P_O2_ changes were also measured in the gas phase inside the well. To that purpose, the sensor was previously calibrated using 0 and 21% O_2_ gas sources. Temperature was continuously monitored with an external temperature sensor immersed in the corresponding medium (gas or liquid).

The second method was based on changes in the fluorescence emission of oxygen-sensible meso-tetrakis(pentafluorophenyl)porphine (PtTFPP) dye (Frontier Scientific Inc, Logan, UT) embedded in a thin PDMS membrane bounded over the membrane of the chip (Thomas et al., 2009). Briefly, 40 mg of PtTFPP in 2.5 mL of toluene (Chromasolv, Sigma-Aldrich, St. Louis, MO) was thoroughly mixed in 5 g of 10:1 PDMS. A 30 μm thick membrane of this mixture was produced as described above by spinning at 1000 RPM for 60 s and baking at 65°C for 2 h for toluene evaporation and PDMS polymerization. Fluorescence quenching of PtTFPP follows the Stern-Volmer equation:

(1) $$\begin{array}{l} \frac{I_{0}}{I}=1+K_{q}\cdot \left[O_{2}\right] \end{array}$$

where *I*_0_ and *I* are fluorescence intensities in the absence and presence of *O*_2_, respectively, *K*_q_ is the quenching constant and [*O*_2_] is *O*_2_ concentration. PtTFPP fluorescence imaging was performed with an inverted microscope (Eclipse Ti, Nikon Instruments, Amsterdam, Netherlands) equipped with a CCD camera (C9100, Hamamatsu Photonics K.K., Hamamatsu, Japan). PtTFPP was excited at the UV-region and emission was captured at 400 nm with a 10 × (0.3 NA) objective. Fluorescence intensity images when applying different oxygen concentrations (0, 5, 10, 15, 20, and 25%) to the chip were processed with ImageJ software (http://imagej.nih.gov/ij/). Data were fitted with Equation (1) to determine *K*_q_. Subsequently, rise time to step changes of P_O2_ (0–20% *O*_2_, 30 s each) was computed.

### Assessment of membrane strain

For strain measurements, a pressure sensor (Honeywell, Morristown, NJ,) was connected to the well and gas input flow was adjusted to generate well-pressures ranging from 0 to 3 kPa. The vertical deflection of the center of the clamped circular PDMS membrane (*h*) was measured by phase contrast imaging with the inverted microscope using a 10 × objective. Assuming that the clamped membrane inflates as a spherical cap, membrane deformation at the well-center is equibiaxial with a linear strain (ε; Winston et al., 1989):

(2) $$\begin{array}{l} \varepsilon =\frac{2}{3}\cdot {\left(\frac{h}{r}\right)}^{2} \end{array}$$

where *r* is the radius of the well. Linear strain in the radial direction varies only slightly across the membrane. However, circumferential strain decreases parabolically to zero at the clamped edge (Williams et al., 1992). Therefore, cells adhered to the central region of the membrane are subjected to a relatively uniform equibiaxial strain, while cells close to the well-periphery deform with an asymmetrical pattern.

Membrane strain was also measured at the center of the well from the displacement of polystyrene microbeads 4.9 μm in diameter (microParticles GmbH, Berlin, Germany) adhered onto the surface of the membrane. Phase contrast images of beads were recorded at different well-pressures and the position of the centroid of the beads was computed with ImageJ. Strain was computed from the relative displacement of beads located at the center of the deflected membrane and compared to that obtained from Equation (2).

To study potential aging of membrane elasticity, a chip was continuously subjected to high amplitude cyclic stretch (ε = ~20%) at 1 Hz for 5 days in 1 × PBS. Membrane strain was measured (Equation 2) with the chip immersed in 1 × PBS and kept at 37°C to mimic conventional culture conditions. Triplicate measurements of membrane pressure-deflection relationship were performed each 24 h.

### Cell-based experiments

The study was performed on rat Lewis bone marrow-derived MSCs kindly provided by Tulane Center of Gene Therapy in New Orleans, LA (Javazon et al., 2001). Cells were cultured in MEM-α medium without ribonucleosides and desoxyribonucleosides (GIBCO, Gaithersburg, MD,) supplemented with 20% fetal bovine serum (FBS; Hyclone Laboratories Inc., Victoria, Australia), penicillin-streptomycin (at concentrations of 100 U/ml and 100 μg/ml, respectively), and 25 ng/ml amphotericin B (Sigma-Aldrich). Prior to cell culture, the surface of the chip membrane was activated with the corona treater by applying the highest voltage during 30 s. Subsequently, the chip was UV-sterilized in a culture hood for 15 min and surface-coated with 0.1 mg/ml collagen type I from rat tail (Cultrex, Trevigen Inc., Gaithersburg, MD; Figure 2E). The volume of collagen solution was adjusted to cover only the surface of the clamped membrane (~30 μl), and coating was performed at 37°C for 30 min (Figure 2F). Membrane surface was washed twice with 1 ml of 1 × PBS and air-dried for ~1 min. Subsequently, a suspension of ~2500 cells in ~10 μl of cell medium was added at the center of the collagen-coated membrane. Cells were allowed to settle and adhere to the membrane for 1 h in a standard cell culture incubator (humidified air, 20% O_2_, 5% CO_2_, 37°C). Then, 4 mL of cell culture medium were added to a culture dish and it was covered with the lid containing the chip (Figure 2G). Cells were cultured overnight in a standard incubator. For the experiments, the chip was placed on the stage of the inverted microscope enclosed in a temperature and humidity-controlled chamber to maintain cell culture at 37°C. The chip was connected to the system described in Figure 1 to subject the cells to the desired gas changes and mechanical strain. Three runs of experiments were performed. In each run, MSCs cultured on five chips were subjected for 4 h to one of the following conditions: normoxia (20% O_2_), SH (1% O_2_), IH (1–20% O_2_, 60 cycles/h, 30 s per O_2_ level), normoxia + stretch, or IH + stretch. Amplitude and frequency of stretch (ε = 6%, 1 Hz) simulated those occurring in the aorta (Goergen et al., 2007).

### Immunochemistry

Immunochemistry at the end-point of experiments was performed as described previously (Garreta et al., 2014). Briefly, cells were fixed in 4% paraformaldehyde for 15 min and non-specific unions were prevented using blocking solution for 45 min. Rabbit anti-HIF-1α (Novus Biologicals, Cambridge, UK) was used as primary antibody. After overnight incubation at 4°C with primary antibody, samples were washed and incubated with anti-rabbit (goat)-Alexa 488 (Jackson ImmunoResearch Laboratories Inc., West Grove, PA) as secondary antibody for 2 h at 37°C. Cellular nuclei were stained with DAPI (Sigma-Aldrich). Epifluorescence images were acquired on the central region of the clamped membrane withthe inverted microscope using a 20 × Plan Fluor Multi-immersion objective (0.75 NA). Images belonging to the same run were collected in a single imaging session using same exposure and illumination settings. For nuclear imaging, samples were illuminated at 330–380 nm and fluorescence emission was recorded using a low pass filter with 420 nm cutoff. HIF-1α fluorescence images were acquired at 515–555 nm when excited at 460–495 nm.

### Image processing

Three images per condition and run of experiment were randomly selected and processed by ImageJ following a blind procedure. HIF-1α nuclear-cytoplasmic fluorescence intensity ratio for each individual cell was determined as previously described (Groulx and Lee, 2002), with some modifications. Total cellular fluorescence of HIF-1α was measured by adjusting Huang thresholding to subtract background and applying a watershed segmentation to delimit cells. HIF-1α fluorescence intensity in nuclear regions was computed from the nuclear outlines previously identified in DAPI channel by adjusting Otsu thresholding. Cytoplasmic HIF-1α fluorescence intensity was obtained by subtracting the nuclear signal to the total cellular signal. Finally, HIF-1α nuclear signal was divided by the cytoplasmic signal to compute nuclear-cytoplasmic fluorescence intensity ratio. The mean ratio of each image was calculated from that obtained from all cells. Then, the mean of the three images belonging to the same run of experiment was also calculated. Subsequently, the differences between experimental conditions from three independent runs of experiments were assessed. Cells in division, out of focus, or not correctly delimited were discarded.

### Statistics

Data are reported as mean ± *SE*. Strain is expressed as percent changes (ε × 100). Differences in means between experimental conditions were analyzed using *t*-tests. Interaction between the effect of gas composition and strain was assessed with two-way ANOVA. Differences were considered significant for *P* < 0.05. Statistical analysis was performed with SigmaPlot (Systat Software, San Jose, CA).

## Results

Figure 2H shows a chip produced with a well of 4 mm in ID and 8 mm in depth and a PDMS membrane 10 μm thick placed in a 35 mm glass bottom culture dish. Inlet (0.3 mm ID, 1 m long), outlet (0.3 mm ID, 20 cm long), and venting (1.3 mm ID, 80 cm long) tubes were connected to the well. To improve cell culture imaging with an inverted microscope, the chip was bounded to the lid of the culture dish. Variations in membrane thickness are negligible (CoV = *SD*/mean = 4%) as assessed by a profilometer.

### Gas equilibrium time through the membrane

**Figure 3:**
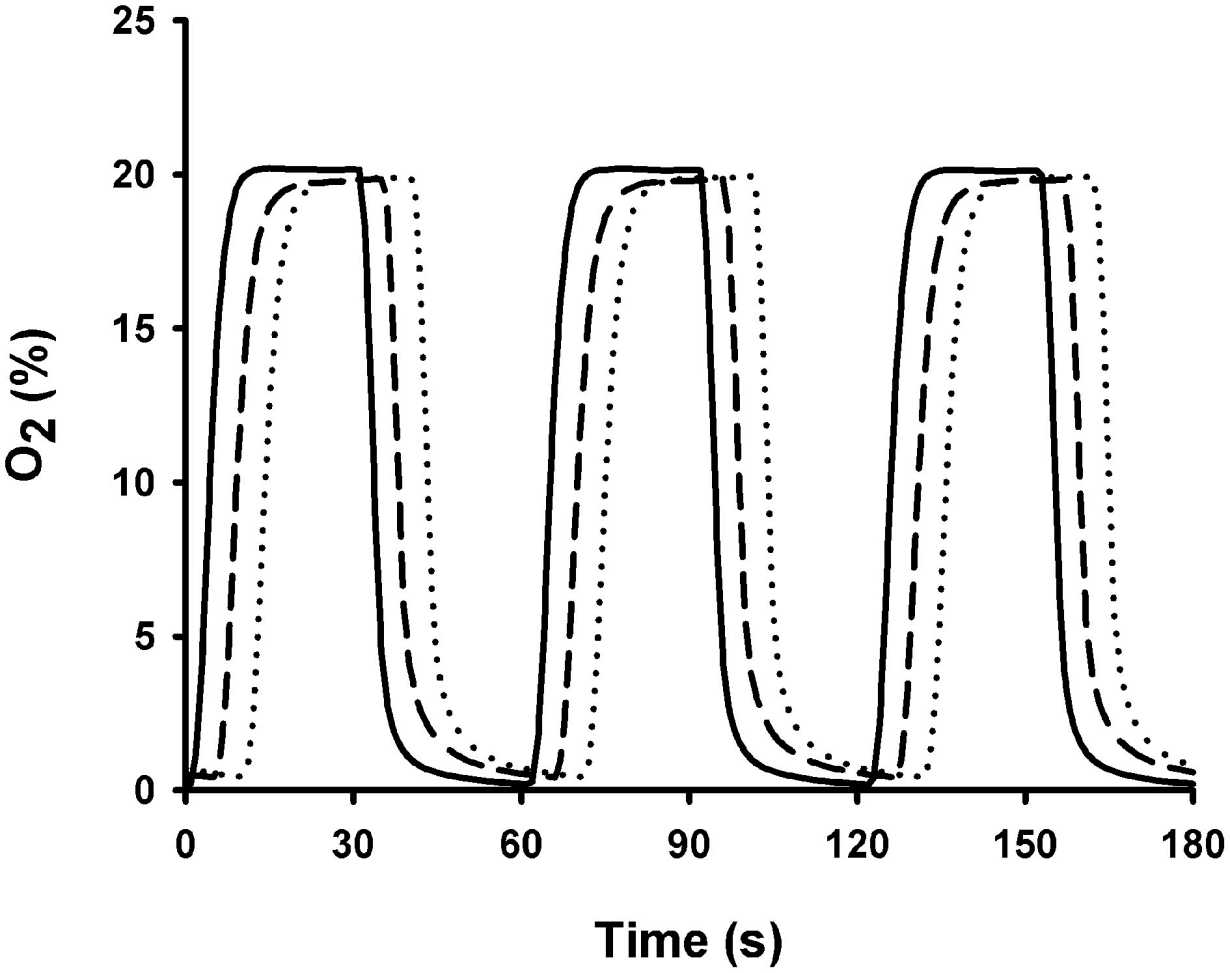
**Oxygen profiles measured below and above the PDMS membranes using a fiber-optic oxygen meter**. P_O2_ was measured above the 10 μm thick (dashed line) and the 37.5 μm thick commercially available membranes (dotted line) when cyclic changes in O_2_ concentration mimicking OSA (30 s at 0% O_2_ and 30 s at 20% O_2_; solid line) were applied to chips. Plots were shifted by 5 s to facilitate comparisons.

The chips were able to mimic patterns of IH associated with OSA. Figure 3 depicts a representative example of P_O2_ changes measured with the fiber-optic oxygen sensor placed at the cell culture level when a gas flow of 30 ml/min with hypoxic-normoxic cycles mimicking OSA (20–0% O_2_, 30 s per condition) was applied to a well 4 mm in diameter and 8 mm in height. Measurements were performed under no stretch conditions. The rise time of P_O2_ change measured on a chip with 10 μm thick PDMS membrane fabricated by spinning was 5.47 ± 0.01 s. These results are in agreement with those derived from PtTFPP fluorescence emission measurements (rise time 5.80 ± 0.39 s). Chips fabricated with 37.5 μm commercially available PDMS membranes (Gel-Pak) exhibited similar rise time (5.34 ± 0.01 s; Figure 3). The rise time computed from P_O2_ measured within the well (5.03 ± 0.01 s) was close to those measured over the membranes. We assessed the homogeneity of IH on the membrane by measuring P_O2_ cycles with the fiber-optic oxygen sensor placed at different radial distances from the well-center. We found uniform P_O2_ swings within the clamped region of the membrane (CoV = 0.5%). However, P_O2_ swings were negligible beyond the well-circle.

### Membrane strain

**Figure 4:**
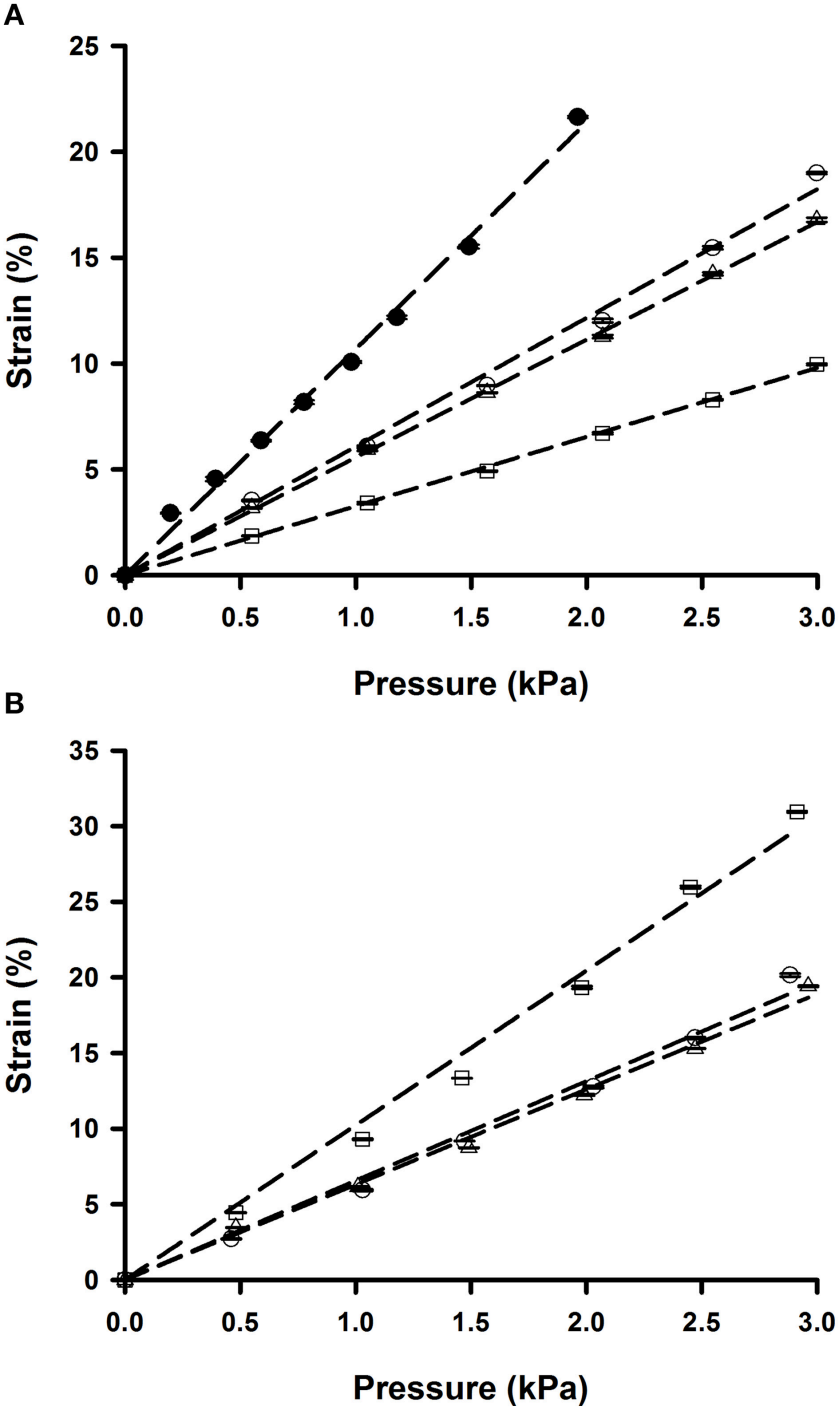
**Pressure dependence of membrane strain computed from central deflection of the membrane. (A)** Chips fabricated with 10 μm thick spun membranes and wells of 4 mm (open symbols) and 8 mm (solid symbols) in diameter. **(B)** Chips with 4 mm wells and commercially available 37.5 μm thick membranes. Data are mean ± *SE* of 3 repeated measurements in each chip. Error bars are smaller than symbol size. Dashed lines are linear fits.

**Figure 5:**
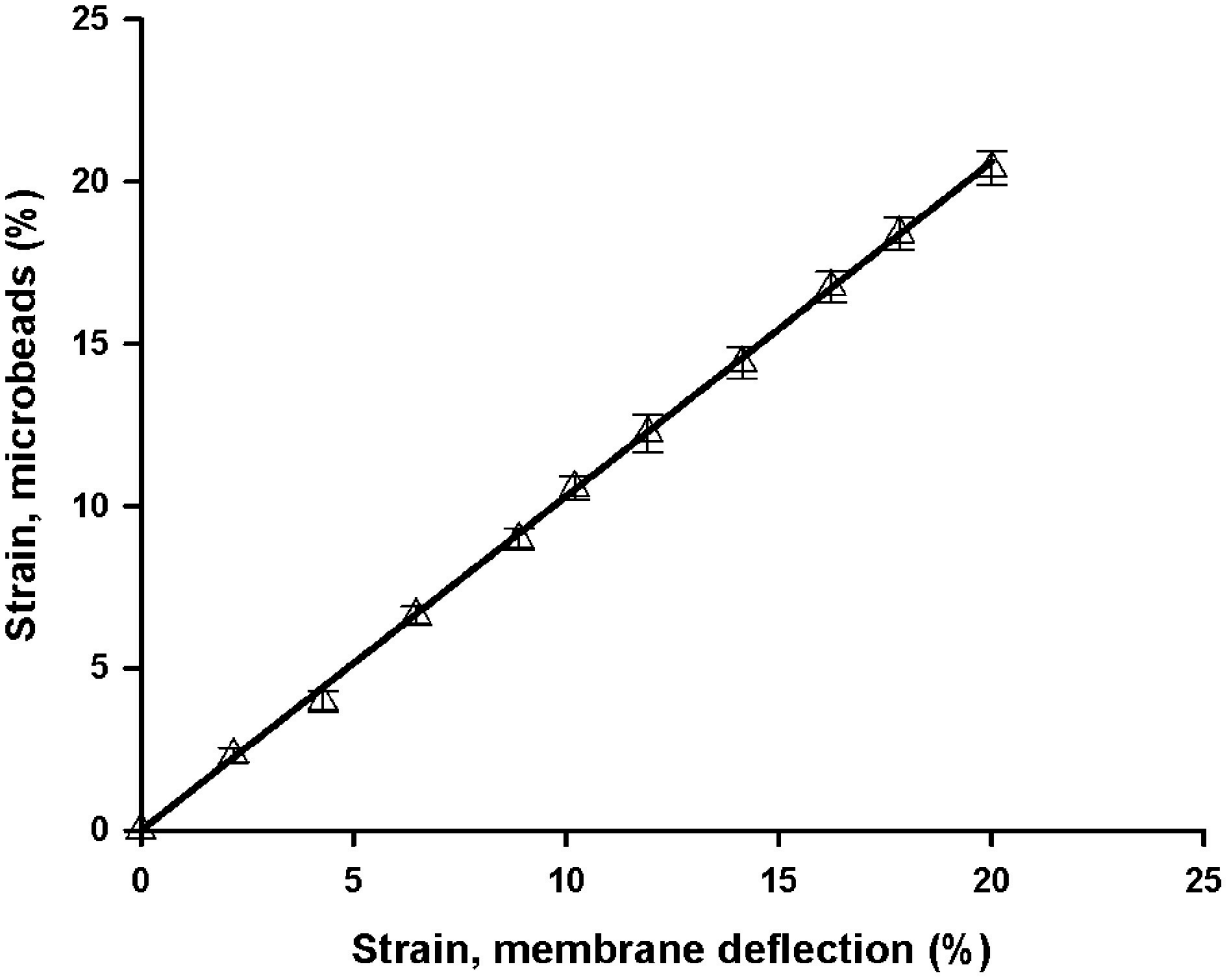
**Comparison of two methods of membrane strain measurement**. Strain was measured in a chip with a 10 μm thick membrane and a well of 8 mm ID from membrane central deflection (Equation 2) and from microbeads attached to the membrane (linear regression slope = 1.03, *R*^2^ = 0.999). Data are mean ± *SE* of three repeated measurements. Error bars are smaller than symbol size.

Membrane strain computed from membrane deflection (Equation 2) increased fairly linearly in the range of well-pressures applied (0–3 kPa; Figure 4). However, the slope of the strain-pressure relationship varied among chips owing to differences in PDMS Young's modulus or membrane thickness. Consequently, each chip must be calibrated prior to experiments. The Young's modulus of the spun and Gel-Pak membranes were 1.65 and 0.29 MPa, respectively. However, the slope of the 37.5 μm thick membranes was only ~50% steeper than that of the 10 μm thick ones (Figure 4), indicating that the ~5-fold higher stiffness of the spun membranes is mostly counterbalanced by their lower thickness (Winston et al., 1989). We also measured the strain-pressure relationship of a chip produced with a well of 8 mm in diameter covered with a 10 μm membrane (Figure 4A). The 2-fold increase in radius resulted in a similar rise in strain-pressure slope. Strain computed from the vertical deflection of the membrane was in close agreement with strain measured from the displacement of microbeads attached to the membrane (Figure 5), showing that membrane strain can be reliably computed from deflection measurements (Equation 2), thereby simplifying the calibration procedure of each chip.

**Figure 6:**
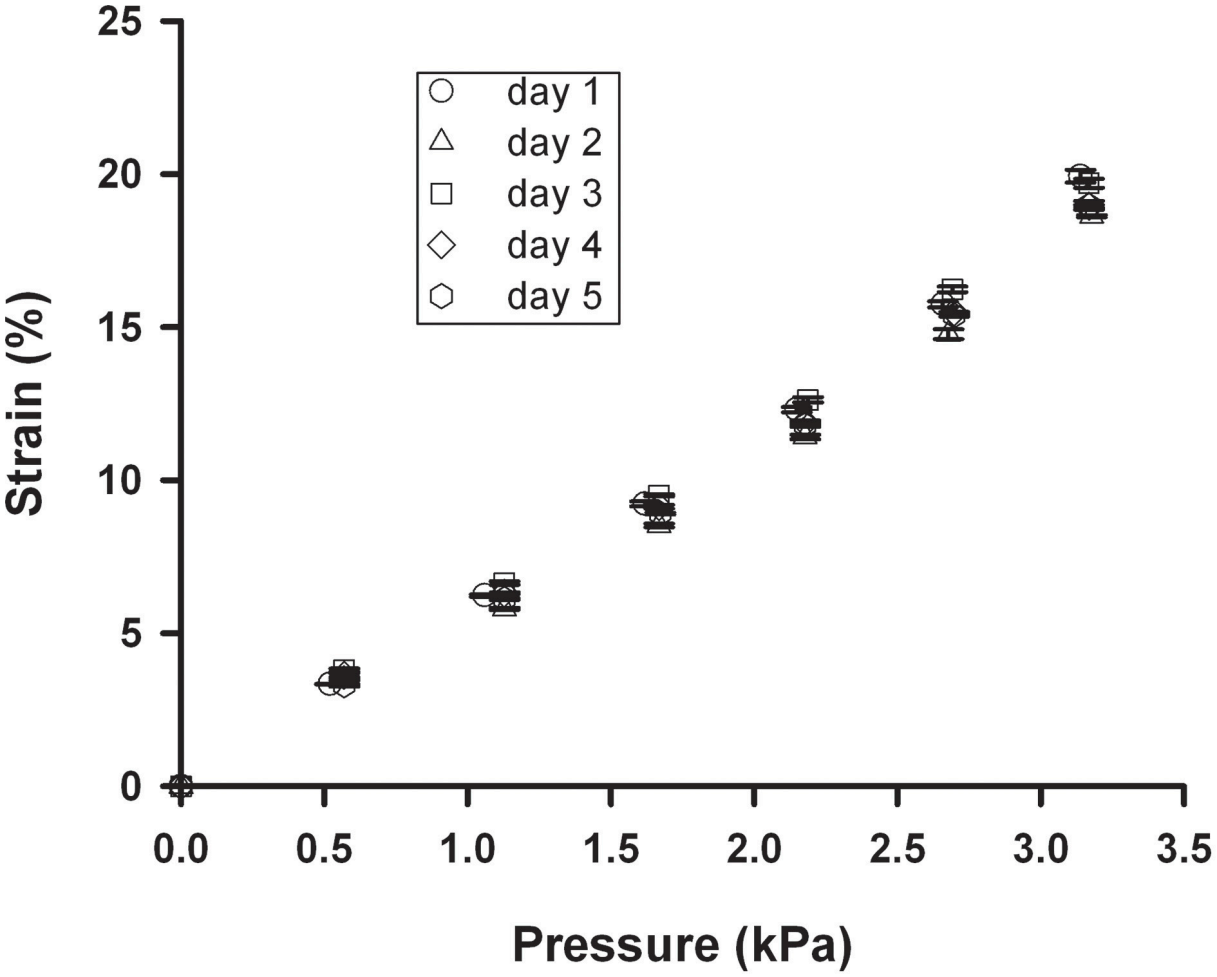
**Strain stability of the membrane**. Strain-pressure relationship measured on different days when 20% strain at 1 Hz was continuously applied to a chip with a 4 mm ID well and a 10 μm thick spun membrane. Data are mean ± *SE* of three repeated measurements.

The PDMS membrane exhibited no aging effects since minimal changes in the strain-pressure relationship were observed after subjecting a chip to 20% strain at 1 Hz for 5 days (Figure 6). Therefore, the PDMS membrane maintains its stiffness and elasticity over time and could be used for long periods without sign of deterioration.

**Figure 7:**
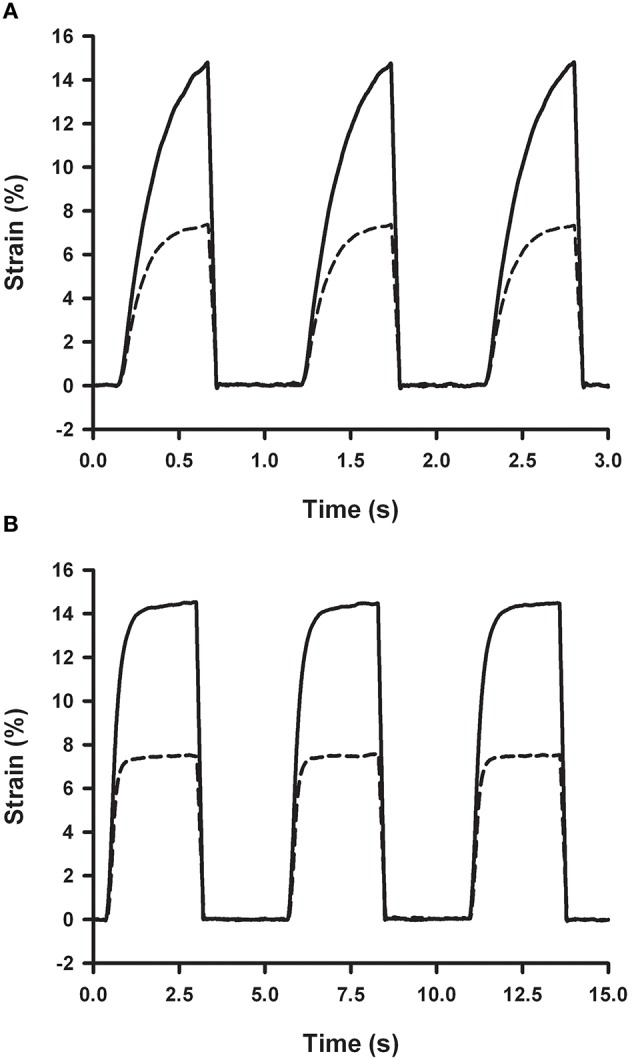
**Strain dependence on outlet resistance and frequency**. Membrane strain measured in a chip with a 4 mm ID well and a 10 μm thick spun membrane with 20 cm (solid line) and 10 cm (dashed line) of 0.3 mm ID outlet tubes obtained with an inlet flow of 30 ml/min (30 s at 0% O_2_ and 30 s at 20% O_2_) by cyclic activation of the two-way venting valve at 1 Hz **(A)** and 0.2 Hz **(B)**.

Cyclic strains at cardiac (1 Hz) and respiratory (0.2 Hz) frequencies were produced by periodic activation of the two-way solenoid valve (Figure 7). Time course of membrane strain was computed by measuring the pressure inside the well and using the strain-pressure relationship of the chip measured under static conditions (Figure 4). Accordingly, peak pressures required to achieve strain amplitudes of 7 and 15% in a 4 mm ID well were ~1.2 and 2.5 kPa, respectively (Figure 4). Since gas flow in the outlet tube has a laminar regime, 2-fold lower well-pressure was obtained by 2-fold shortening of the outlet tube.

### Effect of IH and stretch on HIF-1 expression of MSCs

**Figure 8:**
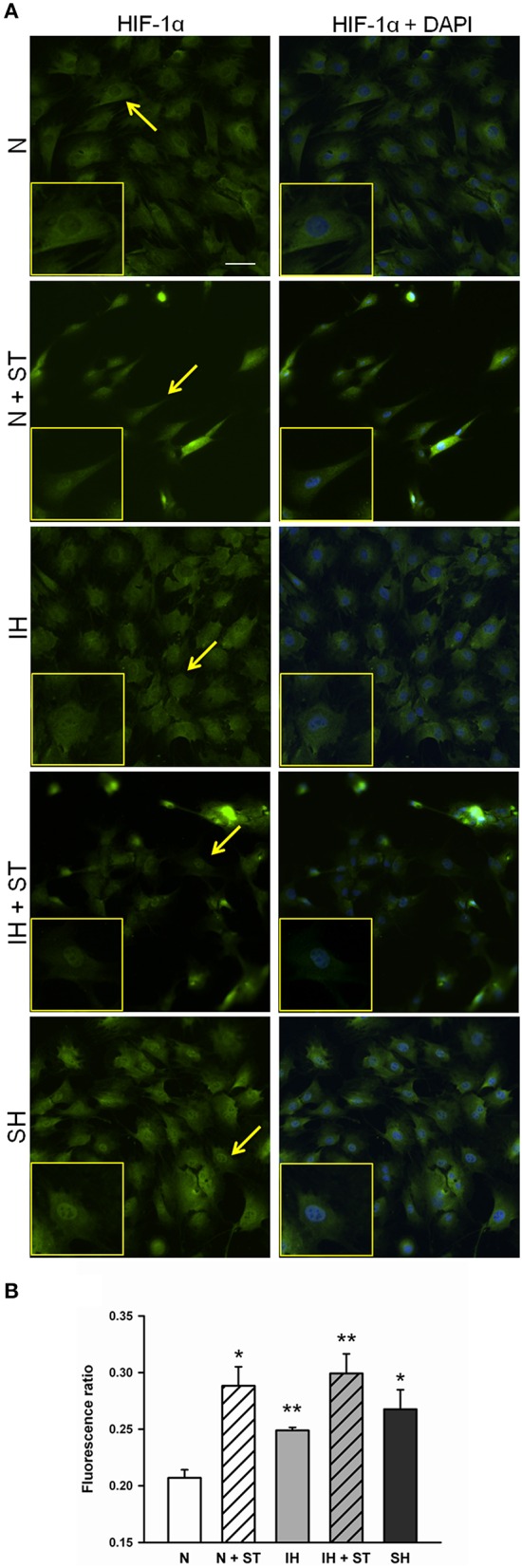
**Effect of P_*O2*_ and stretch on HIF-1α translocation in MSCs**. **(A)** Epifluorescence images of HIF-1α (green, left) and merged (right) with DAPI staining for nuclei (blue) of MSCs after being subjected for 4 h to normoxia (N, 20% O_2_), cyclic intermittent hypoxia (IH, 1–20% O_2_, 30 s per condition), and sustained hypoxia (SH, 1% O_2_) under static conditions, and normoxia (N + ST) and intermittent hypoxia (IH + ST) under cyclic strain (6%, 1 Hz). Insets are zooms of the cells marked with arrows. Scale bar is 50 μm. **(B)** Nuclear/cytoplasmatic ratio of HIF-1α fluorescence (mean ± *SE*). ^*^p < 0.05 and ^**^p < 0.01 for *t*-tests vs. normoxia. *n* = 3 images analyzed per sample.

HIF-1 activation mainly relies on the α subunit and occurs at different levels, including HIF-1α protein stabilization and nuclear translocation, both of which being induced by isolated hypoxia and cell stretch. As HIF-1α nuclear translocation could be considered a qualitative indicator of HIF-1 activity, HIF-1α protein localization was analyzed by immunofluorescence in cells subjected to normoxia, IH, and SH under static conditions, and to normoxia and IH superimposed to cyclic stretch. Representative immunofluorescence images of HIF-1α distribution are shown in Figure 8A. Minimal nuclear signal is observed in normoxic cells whereas the highest signal remains cytoplasmic. On the other hand, HIF-1α nuclear signal seems to be enhanced in cells exposed to hypoxic conditions or cyclic stretch under normoxia or IH. Images of stretch experiments (N + ST and IH + ST) show lower cell density because some cells detached when stretch was applied. HIF-1α nuclear-cytoplasmic fluorescence intensity ratios depicted in Figure 8B show a significant accumulation of HIF-1α in nuclear regions in all conditions when compared to normoxia. The highest nuclear-cytoplasmic ratio was obtained in cells simultaneously subjected to IH and cyclic stretch. No significant interactions were found between IH and cyclic strain (ANOVA, *p* = 0.26).

## Discussion

We describe a low-cost and easy fabrication chip devised to investigate cellular responses to fast changes in gas composition typical of OSA. Cyclic cell stretch at cardiac or breathing frequencies can be superimposed by regulating gas flow pressure within the well. Moreover, the chip is compatible with inverted microscopy. Acute IH mimicking OSA induced HIF-1α translocation from the cytoplasm to the nucleus in MSCs. Cyclic cell stretch at cardiac frequencies also resulted in HIF-1α nuclear translocation.

Our experimental system solves previous technical problems aimed at recreating *in vitro* fast-rate IH and cyclic cell stretch. The performance of the system is based on fast gas diffusion through a thin PDMS permeable membrane separating gas and liquid phases, thereby avoiding long equilibration times (>3 h) due to low oxygen diffusion across culture medium (Allen et al., 2001). The small differences we found in the equilibrium time between measurements taken over and underneath the membranes indicate that the gas equilibration time across the membranes is very short (< 0.4 s). Therefore, the equilibrium time of the whole device (~6 s) is mainly due to the gas composition changes provided by the gas source. Nevertheless, gas composition changes are fast enough to recreate the intermittent hypoxia patterns characteristics of OSA (Ruehland et al., 2009; Lloberes et al., 2011). The equilibration time of our device is lower than commercial and custom-built systems (Gozal et al., 2005; Yuan et al., 2008; Oppegard et al., 2009; Tsapikouni et al., 2012) previously designed to expose cultured cells to fast changes in gas composition. On the other hand, systems currently available with precise temporal or spatial control of P_O2_ require complex processes of fabrication and operation (Acosta et al., 2014; Erickstad et al., 2015) are not well-suited for cell imaging (Baumgardner and Otto, 2003), or are fraught with the need to superimpose shear stress (a considerable confounding factor in inducing cell responses) to IH (Baumgardner and Otto, 2003; Tsapikouni et al., 2012). The design of the current system avoids all these drawbacks. Given that the cell substrate can be cyclically stretchable, the chip can be employed to study the combined effect of IH and cyclic strain present in arterial and lung parenchymal cells. As far as we know, no current device enables simultaneous application of both stimuli. Interestingly, the dimensions of our device enable cell imaging with a conventional fluorescence inverted microscope and thus *in vitro* studies of cellular responses to IH and cyclic strain (e.g., cell migration, proliferation and stiffness, changes in protein expression, or localization).

Inflation of a clamped elastic circular membrane is a common procedure to expose culture cells to cyclic stretch (Winston et al., 1989; Williams et al., 1992; Waters et al., 2012). We fabricated chips with a well of 2 mm radius and a 10 μm thick membrane to subject cells to 6% cyclic strain. Generation of this strain requires a membrane deflection of 0.6 mm (Equation 2) generated by an inflation pressure of ~1 kPa (Figure 4A). Given the laminar flow conditions in the tubing, inflation pressure is proportional to the gas flow, and the resistance of the outlet tubing. Therefore, the strain range can be easily adjusted by modifying the magnitude of the flow provided to the chip or the length or diameter of the outlet tube. Owing to the variability of membrane distensibility, each chip requires stretch calibration. However, this can be performed with a simple one-point calibration by measuring the deflection of the center of the membrane with a phase contrast microscope and adjusting the length of the outlet tubing to achieve the required deflection (Equation 2). We have designed a chip of a small radius that is well-suited for microscopy imaging. However, the amount of biological material available in a well of this size could be insufficient for genetic or protein analysis based on RT-PCR or western blot techniques. Nevertheless, the size of the well can be easily scaled up by increasing the radius of the well and by reducing the deflection pressure (Figure 4A). It should be noted that cells adhered to the membrane away of the well are not subjected to the same P_O2_ swings. We limited the cell culture to the well-opening by restricting ECM coating to the circular clamped membrane, where P_O2_ changes are transmitted uniformly. Alternatively, cell culture area can be restricted by attaching a second PDMS well-over the clamped membrane. We fabricated chips for cell studies by spinning very thin (~10 μm) PDMS membranes to optimize gas exchange kinetics and to minimize the pressure required to inflate the membrane. Moreover, the use of non-humidified gas sources together with low pressures minimizes the risk of air bubble formation in the culture medium. These membranes can be easily produced in conventional soft lithography facilities. Fabrication can be further simplified by using commercially available 37.5 μm thick membranes. Moreover, membranes can be firmly bound to the well-body by PDMS activation with a portable low-cost corona treater. Therefore, fabrication of the chip with commercial PDMS membranes can be carried out with low-cost equipment easily available in most labs.

There is increasing evidence that IH induces inflammation and structural vascular changes in the aorta (Gaisl et al., 2015). In particular, IH is able to promote proliferation of resident macrophages and recruitment of monocytes from bone marrow (Gileles-Hillel et al., 2014). However, the inflammatory response can be counterbalanced by homeostatic mechanisms mediated by adult stem or progenitor cells (Gharib et al., 2010, 2011; Gileles-Hillel et al., 2014). Carreras et al. (2009, 2010a) found in a rat model of OSA that MSCs were mobilized in response to acute IH and that their transplantation was able to reduce IH-induced inflammation. Moreover, MSCs exposed to serum from rats subjected to IH increased their motility and adhesion onto endothelial cells *in vitro* (Carreras et al., 2010b). The repairing role of MSCs in the vascular tissue could be mediated by their capacity for homing to the endothelium and differentiation into different cell phenotypes, as well as by their antioxidative (Kim et al., 2008; Ayatollahi et al., 2014) and immunosuppressive roles ultimately attenuating the inflammatory response (Klinker and Wei, 2015). Although the protective behavior of MSCs seems to be mediated by the activation of HIF-1 signaling pathways (Rey et al., 2009), the molecular mechanisms underlying these processes remain poorly understood. Our *in vitro* model shows that both IH and cyclic stretch promote HIF-1 activation in MSCs. Despite that both stimuli induce the activation of HIF-1α, the mechanisms involved are presumably distinct. While low oxygen tensions inhibit the degradation of HIF-1α via PHDs, mechanical stress-mediated HIF-1α induction seems to rely on the activation of distinct ion-dependent signaling cascades such as the phosphatidylinositol 3-kinase (PI3K)/protein kinase B (Akt)/FKBP-rapamycin-associated protein (FRAP) pathway or the Yes-associated protein (YAP)/transcriptional co-activator with PDZ-binding motif (TAZ) via stretch-sensitive ion channels (Kim et al., 2002; Milkiewicz et al., 2007; Bendinelli et al., 2013; Wagner et al., 2013). Taken together, our results support the notion that both IH and cyclic stretch could participate in the homeostatic response mediated by MSCs and highlight the importance of integrating both stimuli when evaluating OSA consequences on tissues such as arterial endothelium or lung parenchyma.

Although the design of our chip was aimed at investigating the molecular mechanisms leading cellular responses to cyclic stretch and IH mimicking OSA, its application could be extended to a wide variety of research areas due to its versatility and simplicity of fabrication and handling. One area of special research interest is cancer. In fact, SH and IH are conditions normally found in solid tumors and many of the adverse outcomes of cancer (e.g., higher risk of metastasis) are mediated by HIF-1 signaling pathways (Vaupel and Mayer, 2007; Bristow and Hill, 2008). In addition, given the high gas permeability of PDMS, the chip is also able to provide chronic and intermittent hypercapnia conditions associated with several pulmonary diseases such as chronic obstructive pulmonary disease. Additionally, the adverse consequences of treatments such as mechanical ventilation can be studied by exposing cells to high stretch and hyperoxia.

Notwithstanding, the current device presents some potential limitations. The first one is associated with the non-uniform deformation of the circular clamped membrane, a problem that is in common with other stretchable devices including commercially available systems such as Bioflex culture plates (Flexcell International Corporation, Burlington, NC), which are based on the inflation/deflation of circular elastic diaphragms. Although in these systems lineal strain slightly varies across the membrane in these systems, the circumferential strain decreases parabolically to zero at the edges of the circular clamped membrane, thereby resulting in non-uniform strain exposures of cells cultured on different radial positions from the center of the membrane (Williams et al., 1992). Accordingly, the interpretation of the results should be restricted to the central region of the membrane, where cells experience a uniform equibiaxial strain. Alternatively, the geometry of the membrane deformation could be switched from spherical to cylindrical based on the fabrication of rectangular wells instead of circular wells (van Rijn, 2004). On the other hand, the current design does not provide shear stress simultaneous to O_2_ tension control and cyclic stretch. However, this limitation could be easily overcome with a minor modification in the design of the system to allow the continuous inflow of cell culture medium from a reservoir. Therefore, the incorporation of simple modifications would provide more opportunities for using this chip to respond to other relevant questions, with particular interest in the field of human vascular disease and cancer. Finally, the IH amplitude of the P_O2_ changes employed for the validation of the chip was chosen in such a way to maintain consistency with previous studies (Ryan et al., 2005; Yuan et al., 2008). However, the amplitude and frequency of the P_O2_ changes could be adjusted to more physiologic values in future experiments due to the versatility of the chip.

In conclusion, the present chip design recreates *in vitro* the hypoxia-re-oxygenation patterns characteristics of OSA, thereby offering a novel and readily implementable approach permitting investigation of the cellular effects of OSA, in particular in the arterial endothelium and lung parenchyma. Here, by using this novel experimental approach, we have shown that IH as well as stretch promote HIF-1 activation on MSCs. The versatility of combining different experimental conditions and cell types, and its low-cost and easy fabrication could extend the use of the chip to other laboratories and fields of research.

## Author contributions

NC, DG, RF, IA, and DN participated in the conceptual framework of the project. NC, IJ, LS, and BC performed experiments. NC, IJ, DG, RF, IA, and DN analyzed and interpreted experimental data. NC, RF, DG, IA, and DN contributed to the writing of the manuscript. All authors approved the final version of the manuscript.

## Funding

IA was supported by Beatriu de Pinós fellowship (Agència de Gestió d'Ajuts Universitaris i de Recerca) from Generalitat de Catalunya (2010 BP-A2-00023). This work was partially funded by Ministerio de Economía y Competitividad (PI11/00089, PI14-00280 to DN and PI14/0004 to RF) and Sociedad Española de Neumología (SEPAR-086/2014 and 139/2015 to IA).

### Conflict of interest statement

The authors declare that the research was conducted in the absence of any commercial or financial relationships that could be construed as a potential conflict of interest.
