# Supplementary material for: A Novel Chip for Cyclic Stretch and Intermittent Hypoxia Cell Exposures Mimicking Obstructive Sleep Apnea
Source: Front Physiol. 2016 Jul 29;7:319. doi: 10.3389/fphys.2016.00319 (PMC4965455; doi:10.3389/fphys.2016.00319)
Supplement: Supplementary file 2 [file DataSheet1.DOCX]

**SUPPLEMENTARY MATERIAL**

**A novel chip for cyclic stretch and intermittent hypoxia cell exposures mimicking obstructive sleep apnea**

Noelia Campillo^1,2,3^, Ignasi Jorba^1,2,3^, Laura Schaedel^1,2^, Blai Casals^1,2^, David Gozal^4^, Ramón Farré^1,3,5^, Isaac Almendros^1,3,5^, Daniel Navajas^1,2,3^

**Step-by-step protocol for membrane and chip fabrication**

**MATERIALS**

**Reagents**

- Acetone (Panreac AppliChem, cat. no. 211007.1214)
- Isopropyl alcohol (Panreac AppliChem, cat. no. 3465,1000)
- Methanol (Panreac AppliChem, cat. no. 141091.1214)
- Ethanol absolute (VWR International, cat. no. 83813.360)
- Repel Silane (Tridecafluoro-1,1,2,2-tetrahydrooctyl)-trichlorosilane) (ABCR GmbH & Co. KG, cat. no. AB111444). **CAUTION:** *Repel Silane is considered toxic and should be manipulated inside a hood, using the adequate laboratory safety equipment and discarded as hazardous waste.*
- 37.5 µm thick Gel-Pak membranes (Gel-Pak, cat. no. PF-60/1.5-X4)
- Polydimethylsiloxane (PDMS) (Dow Corning, Sylgard 184 kit, cat. no. 01064291)

**Equipment**

- 2 Bell jar vacuum desiccators (Kartell Labware, cat. no. 554). **CAUTION:** *one desiccator is exclusively used for surface exposures to Repel Silane toxic vapors and is enclosed in a hood. The other desiccator is only used to degas PDMS*
- Portable corona treater (Electro Technic Products, BD-20AC model)
- Plasma cleaner (Harrick Scientific Products Inc., PDC-002 model)
- Spinner (Laurell Technologies Corporation, WS-650MZ-23NPP/LITE model)
- Precision balance (A&D Instruments Ltd, EJ-200 model)
- Hot plate (Selecta, 6155100 model)
- Oven (Selecta, 2000200 model)
- 0.3 mm ID polytetrafluoroethylene tube (Cole Parmer, cat. no. 06417-11)
- 1.3 mm ID polytetrafluoroethylene tube (Cole Parmer, cat. no. 06417-51)
- 1 mm diameter punch (Harris Uni-Core, cat. no. 15099)
- 2 mm diameter punch (Harris Uni-Core, cat. no. 15076)
- 4 mm diameter punch (Harris Uni-Core, cat. no. 15080)
- 0.5-10 µl micropipette (Eppendorf, cat. no. ES-10)
- 10 µl pipette tips (DasLab, cat. no. 1001807L)
- 76 x 26 mm glass slide (Deltalab, cat. no. D100002)
- Borosilicate dishes (Duroplan, cat. no. 2175553). **CAUTION:** *do not use plastic dishes for glass slide cleaning since reagents are corrosive*
- 18 mm diameter coverslips (VWR International, cat. no. 631-0153). **NOTE:** *Other sizes can be alternatively used*
- 150 cm^2^ plastic culture dish (Techno Plastic Products AG, cat. no. 193150)
- 35 mm glass bottom culture dish (MatTek Corporation, cat. no. P35G-1.5-20-C)
- Drill (Comei S.L., C2 model)
- Glass Pasteur pipette (VWR International, cat. no. 612-1702)
- Pressurized N_2_ source
- Plastic Pasteur pipette (Deltalab, cat. no. 200006C)
- Plastic square weigh boat (VWR International, cat. no. 611-0094)
- Scalpel (Swann Morton, cat. no. 0206)
- Tweezers (VWR International, cat. no. 232-0111)

**PROCEDURE**

**Mixing and degassing of PDMS** (•**TIMING** ~50 min)

Prepare and degas a mixture of PDMS silicone elastomer base and curing agent for well molding and also to produce the membrane and assemble the chip.

1. Place an empty plastic weight boat on a precision balance and pour silicone elastomer base and curing agent in a 10:1 (base:curing agent) weight proportion.

**NOTE:** Prepare enough amount (~130 g) to produce a PDMS block with the desired thickness (~8 mm) and to fabricate the membrane.

1. Using a plastic Pasteur pipette, mix vigorously (~1 mm) base and curing agent.

**CAUTION:** It is important to mix the components thoroughly to achieve a proper polymerization and to obtain a homogeneous PDMS block.

1. Pour 120 g of the mixture into a 150 cm^2^ plastic culture dish and leave the remaining PDMS in the dispensable weigh boat for the ulterior PDMS membrane preparation and chip assembly.
2. Place the open dish and the weigh boat containing the PDMS in a vacuum dessicator.
3. Apply vacuum for 45 min to remove air bubbles from PDMS.

**Fabrication of the PDMS well** (•**TIMING** ~ 3 h)

1. Remove the culture dish containing the uncured PDMS from the bell jar dessicator.
2. Cover the culture dish with the lid and place it in an oven at 65ºC for 3 h to cure PDMS.

**TROUBLESHOOTING***

**NOTE:** The dimensions of the obtained block results sufficient to produce ~100 wells.

1. With a scalpel, cut the cured PDMS into rectangular (~10 mm x ~10 mm) blocks.
2. Perforate the block from top to bottom with a 4 mm ID punch to produce a central well.
3. Make a lateral perforation in two opposite sides of the well using a 1 mm ID punch.

**NOTE:** These lateral perforations will be used to connect inlet and outlet tubes to the well as described in steps 41 and 42.

1. Perforate a third lateral side of the block using a 2 mm ID punch, connecting to the well.

**NOTE:** This hole will be used to connect the well to the venting tube to the well as described in step 43.

**Fabrication of the PDMS membrane** (•**TIMING** ~ 2h)

Custom built PDMS membranes can be fabricated by using the PDMS mixture prepared in steps 1-6. Alternatively, commercially available Gel-Pak membranes can be employed and proceed immediately to step 31.

1. Dispose three borosilicate dishes containing 20 ml of acetone, methanol or isopropyl alcohol in a hood.
2. Clean a 76x26 mm glass slide by gentle agitation in each solution for 30 s in a sequential manner (acetone → methanol → isopropyl alcohol).

**NOTE:** Use flat square tip tweezers to facilitate glass slide manipulation and avoid surface contact with gloves.

1. Dry out the glass slide using a stream of compressed N_2_. Alternatively, a source of pressurized air can be also used.
2. Place the slide on a hot plate at 95ºC for 20 min to ensure a complete dehydratation.
3. Dispose the slide in the enclosed chamber of a plasma cleaner.
4. Activate the surface of the slide with oxygen plasma using the highest power (≈18 W) for 30 s.

**NOTE:** This step must be performed with the plasma cleaner instead of the portable corona treatment to obtain a more homogeneous activation of the slide.

1. Turn off the plasma cleaner and remove the glass slide from the chamber.

**CAUTION:** Handle the glass slide with care to avoid touching the activated surface. Proceed immediately to step 18 to prevent time-dependent surface inactivation.

1. Place the wafer in the corresponding desiccator surrounded by two coverslips.
2. Dispense a small drop containing 3 µl of Repel Silane on the coverslips and apply vacuum to induce the release of Repel Silane vapors.

**CAUTION:** The wafer should be maintained in a horizontal position to favor the deposition of vapors emanating from the silanizing agent. Perform this step in a fume hood wearing safety glasses, gloves and a lab coat to prevent hazardous effects.

1. Remove the slide from the desiccator after 1h of silanization.

**NOTE:** Although silanized slides could be stored for a few days without losing their treatment, it is recommended to proceed subsequently to step 22.

1. Remove the boat weigh containing the PDMS mix prepared during steps 1-6 from the desiccator.
2. Eliminate possible residues from the glass slide using a stream of pressurized N_2_ prior to step 24.
3. Place the silanized glass slide on the holder of a spinner and apply vacuum to immobilize.
4. Using a plastic Pasteur pipette, pour PDMS on one edge of the surface of the glass slide were silane vapors were deposited.
5. Extend twice the PDMS from one edge to the other, using a glass Pasteur pipette.

**TROUBLESHOOTING***

**CRITICAL STEP:** Perform steps 25 and 26 carefully, avoiding air bubble formation to obtain homogeneous PDMS membranes.

1. Spin-coat the PDMS for 5 s at 2000 RPM and then for 60 s at 5000 RPM to obtain the desired thickness (10 µm).
2. Disconnect vacuum and remove the glass slide containing the spun PDMS.
3. Place the slide on a leveled hot plate at 95ºC for 20 min to cure the membrane.
4. Remove the wafer from the hot plate and allow it to cool for 5 min.

**NOTE:** The membrane can be stored in a closed culture dish at room temperature until its utilization.

**Chip assembly** (•**TIMING** ~ 4 h)

1. Prior to step 32, remove possible particles that may have been deposited on the surface of the fabricated membrane and the well using compressed filtered N_2_.
2. Activate the PDMS well by applying oxygen plasma at close proximity (~ 5 mm) at the highest voltage using the portable corona treater for 1 min. Proceed immediately to step 33.
3. Activate the fabricated or the commercial membrane applying plasma (~ 5 mm) with the corona treater for 30 s.

**TROUBLESHOOTING***

**NOTE:** If using commercial membranes, peel off the plastic layer protecting the membrane prior to its treatment.

1. Immediately after plasma activation, place in contact the activated surfaces and apply pressure.
2. Dispense uncured PDMS with a 10 µl pipette tip at the edges to facilitate the ulterior handling.
3. Cure on the hot plate at 95ºC for 20 min.

**CAUTION:** When using commercial membranes, interpose a glass slide between the plastic layer supporting the membrane and the hot plate to avoid plastic melting.

1. Cut at the edges using a scalpel and peel off the block containing the membrane from the slide very carefully.

**TROUBLESHOOTING***

**CRITICAL STEP:** To facilitate membrane detachment from its support, add absolute ethanol with a plastic Pasteur pipette between them. This results in the reduction of surface tension whilst peeling.

1. To improve cell culture imaging in an inverted microscope, adhere the base of the device to the lid of a 35 mm glass bottom culture dish by dispensing PDMS around its edges using a micropipette tip.

**TROUBLESHOOTING***

**NOTE:** When using an upright microscope, the chip could be directly adhered to the bottom of the culture dish.

1. Place in the oven at 65ºC for 2 h to achieve the complete adhesion of the chip to the dish.
2. Once the chip is completely adhered, drill the culture dish to allow the passage of inlet, outlet and venting tubes for gas inflow and outflow.
3. Insert a 1 m long 0.3 mm ID in one of the 1 mm holes performed in step 10 (inlet tube).
4. Insert a 10 cm long 0.3 mm ID in the other 1 mm hole performed in step 10 (outlet tube).
5. Insert a ~ 80 cm long 1.3 mm ID tube into the 2 mm hole performed in step 11 (venting tube).

**CAUTION:** Do not press the tubes during their manipulation to prevent their collapse or ulterior changes in resistance.

1. Seal all possible gas and liquid leakages by dispensing PDMS in areas at risk (tube passage).
2. Cure PDMS in the oven at 65ºC for 1 h.
3. Examine the fabricated chip in an optical microscope.
4. Calibrate the chip to ensure its correct operation prior to its use.

**TROUBLESHOOTING TABLE**

| Step | Problem | Possible reasons | Solution/s |
| --- | --- | --- | --- |
| 7 | Irregular block thickness | The surface where PDMS was cured during well molding was not leveled or flat enough | Be sure that the oven is clean and properly leveled |
| 26 | Trapped air bubbles in the fabricated membrane | Air bubbles were generated when PDMS was poured onto the slide prior to spin coating | Pour degassed PDMS carefully onto the slide avoiding bubble formation. If necessary, remove bubbles blowing air using a plastic Pasteur pipette |
| 33,37 | Membrane sags formation | Membrane was overtreated with plasma  The chip was peeled off from its support too fast | Adjust membrane treatment to the time recommended (30 s) and do not exceed it  Peel off the membrane from its support carefully and slowly. Add ethanol to reduce surface tension |
| 37 | Membrane remains adhered to the slide | Glass slide silanization was not performed correctly  Activation of the membrane and well surfaces was not enough | Be sure that the position of the slide in the desiccator is correct (horizontal and surrounded by two coverslips containing Repel Silane drops) and that vacuum is not lost during the exposure (1 h)  Be sure that plasma is applied in close proximity and along all the surface during the time indicated |
| 38 | Surface of the chip cannot be imaged with the microscope | Thickness of the chip is below or above the working distance of the objective employed | Consider the characteristics of the objective that will be employed for strain calibration / live cell imaging and adjust the thickness of the chip accordingly (see steps 1-3) |
